# Complete genome sequence of *Pediococcus pentosaceus* 13.7 13A-2 isolated from a Jersey milk cattle

**DOI:** 10.1128/mra.00847-25

**Published:** 2025-11-05

**Authors:** Sebastian W. Fischer, Nadine Mariani Corea, Jennifer Wachtarczyk, Fritz Titgemeyer

**Affiliations:** 1 Institute for Hygiene and Public Health, University Clinics Bonn, Bonn, Germany; 2 Department of Food, Nutrition, Facilities, FH Münster 39002, Münster, Germany; Fluxus Inc., Sunnyvale, California, USA

**Keywords:** lactic acid bacteria, probiotics, protective cultures, Nanopore, biofilm

## Abstract

We present the genome sequence of *Pediococcus pentosaceus* 13.7 13A-2, isolated from a Jersey dairy cow from a conventionally managed dairy farm in Münsterland, Germany. The circular chromosome comprises 1.77 Mbp and 1,686 predicted protein-coding genes. It provides the molecular basis for investigating its potential application in foods.

## ANNOUNCEMENT

*Pediococcus pentosaceus* is a lactic acid bacterium used to ferment milk, meat, vegetables, and fruits (1, 2). It shows probiotic activity and improves food safety by inhibiting foodborne pathogens (2–4).

The strain was isolated from a swab introduced into the teat canal to obtain the biofilm of the bovine papillary duct from a cow from a conventionally managed milk farm in Münsterland, Germany (GPS: 51.95N 7.63E). The swab was transferred to 1 mL of 0.9% NaCl solution. Serial dilutions were plated onto De Man, Rogosa, and Sharpe (MRS)-Bouillon-agar plates supplemented with 0.5 g/L cysteine and 10 mg/L bromophenol blue and incubated anaerobically at 30°C for 48 h (5). Colonies were clonally isolated and subjected to colony PCR for amplification of an *rrnA* gene fragment using primers 27f (5′-AGAGTTTGATCCTGGCTCAG-3′) and N1492R (5′-TACGGYTACCTTGTTAYGACTT-3′) (6). The Sanger sequenced DNA of 1,101 bp from base 77 to 1,177 was 100% identical to *rrnA* of *P. pentosaceus* ATCC 25745 (NC_008525.1).

The strain was cultured on MRS agar with 0.5 g/L cysteine for 48 h at 30°C. Genomic DNA was isolated using the Wizard HMW DNA Extraction Kit (Promega, USA) and quantified on a DeNovix QFX fluorometer (DeNovix, USA). The sequencing library was prepared using the Oxford Nanopore SQK-RAD004 kit. The kit’s transposase fragmented the genomic DNA, and no additional size selection was performed. The sample was loaded (30 µL) onto a Flongle Flow Cell R9.4.1 (FLO-FLG001) mounted on a MinION Mk1B device running MinKNOW v22.08.9 (7). Raw signals were base-called with Guppy v6.3.8 (high-accuracy model dna_r9.4.1_450bps_hac.cf g) (8). Reads with a mean Phred quality ≥Q9 were retained. Adapter trimming was performed with Porechop v0.2.4 (9). Reads were further filtered with Filtlong v0.2.1 to discard the worst 10% by quality and all reads <1 kb (10). The final data set comprised 59,877 reads (240 Mbp; read N50 = 5,834 bp; median/mean read length = 4,008/2,569 bp; longest read = 53,152 bp; mean read Q = 13.1; 59.2%/16.5% of bases reached ≥Q20/≥Q30). Quality metrics were obtained with Nanoq v0.10.0 and SeqKit v2.9.0 (11, 12). Assembly was managed with a Snakemake workflow that implemented Trycycler v0.5.4. Sub‑assemblies were generated with Flye v2.9.2, Raven v1.8.2, and Miniasm v0.3, followed by MiniPolish v0.1.3; consensus reconciliation used Mash v2.3, minimap2 v2.25, and MUSCLE v3.8 under R v4.3.1 (packages *ape* v5.7‑1 and *phangorn* v2.11‑1) (13–26). The draft consensus was polished with Medaka v1.8.0 and homopolished v0.4.1 in mode polish and modpolish (27, 28). The circular chromosome was re‑oriented to start at *dnaA* using Dnaapler v1.2.0. The correctness was verified with Bandage v0.9.0. (29, 30). Unless otherwise noted, all bioinformatics tools were used with default settings.

QUAST v5.3.0, using *P. pentosaceus* ATCC 25745 (NC_008525.1) as reference, yielded the metrics shown in Table 1; BUSCO v5.8.3 (data set *pediococcus_odb12*) reported 98.80% completeness (31, 32). CheckM2 v1.1.0 (specific neural-network model) confirmed 100% completeness and 0.23% contamination (33). Read depth, obtained by remapping filtered reads with minimap2 v2.30 and samtools v1.22.1, showed a mean coverage of 113.9× across the chromosome (21, 22, 34).

**TABLE 1:** Assembly and quality metrics for the complete chromosome of *P. pentosaceus* 13.7 13A-2

Replicon	Length (bp)	G+C (%)	BUSCO completeness (%)	CheckM2 completeness/contamination (%)	Genome fraction (%)
Chromosome	1,771,625	37.14	98.80	100/0.23	85.61

Genome features were annotated with PGAP v6.10, identifying 1,686 protein-coding sequences, 15 rRNA genes, and 55 tRNA genes (35).

## Data Availability

Whole-genome sequencing data for *Pediococcus pentosaceus* strain 13.7 13A-2 have been deposited in the NCBI Sequence Read Archive (SRA) under BioProject accession number PRJNA1297324. The raw sequencing reads are available via the SRA under the corresponding BioSample AMN50221057 entry. The assembled and annotated genome is available at DDBJ/ENA/GenBank accession numbers JBPWPB000000000, NZ_CM1266291, and GCF_052402805.1. The version described in this paper is version JBPWPB010000000.
